# Effect of the Amount of *Ephestia kuehniella* Eggs for Rearing on Development, Survival, and Reproduction of *Orius laevigatus*

**DOI:** 10.3390/insects13030250

**Published:** 2022-03-01

**Authors:** Francisco Javier Gallego, Amador Rodríguez-Gómez, María del Carmen Reche, Virginia Balanza, Pablo Bielza

**Affiliations:** Departamento de Ingeniería Agronómica, Universidad Politécnica de Cartagena, 30203 Cartagena, Spain; franchiskojavier@hotmail.com (F.J.G.); amador.rodriguez@upct.es (A.R.-G.); mariadelcarmen.reche@edu.upct.es (M.d.C.R.); virginia.balanza@upct.es (V.B.)

**Keywords:** biological control, mass rearing, diet, factitious prey, omnivorous predator, zoophytophagous, cost

## Abstract

**Simple Summary:**

Economical mass rearing of natural enemies is pivotal for their use as augmentative biological control agents. To this end, the conditions for mass production have been widely investigated for different natural enemies, including the predator *Orius laevigatus*. The key component for cost optimization of production systems is the type and amount of diet supplied. In the case of *O. laevig*atus, *Ephestia kuehniella* eggs have proved to be nutritionally superior to other cheaper natural and artificial foods. Consequently, the current practice in industrial production is the use of these eggs as food. Therefore, there is a need to know the minimum amount of *Ephestia* eggs needed per individual leading to a cost reduction, which in turn will favor biological control adoption. In this study, we established a minimum of 1 and 3 *Ephestia* eggs per day for the first two nymphal instars, respectively, and 8 eggs per day for subsequent development until adulthood. Benefits and savings for industrial production of *O. laevigatus* and its use in biological control are discussed.

**Abstract:**

*Orius laevigatus* is a key tool for the success of augmentative biological control programs in protected crops. This biological control agent is mass-reared feeding on eggs of *Ephestia kuehniella*. However, this factitious prey is expensive, accounting for a significant percentage of the rearing costs. Therefore, there is a need to optimize the amount of *Ephestia* eggs needed per individual leading to a cost reduction, which in turn will favor biological control adoption. This study investigated the effect of the amount of *Ephestia* eggs provided on the developmental and reproductive fitness of *O. laevigatus*. At least a daily supply of 1 and 3 *Ephestia* eggs was needed for optimal development of the first two nymphal instars, respectively, although for maximum survival, 1 egg was enough for both instars. For subsequent development until adulthood, a minimum of 8 eggs per day were needed to fully support growth, but only 3 eggs for optimal survival. Similarly, male body size was also maximized by feeding 8 eggs, but for maximum female body size 10 eggs per day were required. Oviposition rate of females increased with the daily number of *Ephestia* eggs provided, until a plateau was reached at 8 eggs/day. Benefits and savings for industrial production of *O. laevigatus* are discussed.

## 1. Introduction

*Orius laevigatus* (Fieber) (Hemiptera: Anthocoridae) is a key tool for the success of augmentative biological control programs in protected crops in Europe, Africa, and Asia [1,2,3]. This generalist predator is widely used to control small pests, especially against thrips such as *Frankliniella occidentalis* (Pergande) (Thysanoptera: Thripidae) [3,4,5,6]. As a biological control agent, it is mass-reared, feeding on eggs of *Ephestia kuehniella* Zeller (Lepidoptera: Pyralidae) (hereafter *Ephestia* eggs). However, this factitious prey is expensive, with market prices varying around 600–800 €/kg [7], accounting for a significant percentage of the rearing costs. Therefore, there is a need to optimize its mass scale production leading to a cost reduction, which in turn will contribute to a wider biological control adoption. Different attempts have been conducted in order to find a cheaper food source, such as pollen, artemia cysts, or several artificial diets [8,9,10,11,12,13,14,15,16,17,18,19,20]. However, no other food provided the quality of *Ephestia* eggs. Another approach is to optimize the amount of *Ephestia* eggs needed per individual for survival and development from egg to adult and for oviposition upon adult emergence.

Studies of the effects of *Ephestia* eggs density on the developmental performance of other *Orius* species has been reported [21,22]. However, those works did not study the effect on each nymphal stage, just for the whole development from egg to adult. Yet, it is expected that younger instars will require less food supply than older (larger) stages. Therefore, fine-tuning the amount of diet for each immature stage will further optimize the production costs.

The current study investigated the effect of the amount of *Ephestia* eggs provided on the developmental and reproductive fitness of *O. laevigatus* in order to assess the optimal number of eggs per day for each life stage for cost-effective mass production.

## 2. Materials and Methods

A commercial population of *O. laevigatus* was purchased from Agrobio SL (Almeria, Spain, ORIcontrol^®^). This population was reared in the laboratory by using 1-L plastic containers with filter paper on the lid, with ad libitum access to frozen *Ephestia* eggs as food, pieces of green bean pods as moisture source and egg-laying substrate, and black wheat husk as hideout to avoid cannibalism. The containers were maintained under controlled conditions at 26 ± 1 °C, 65 ± 5% rh, and L16:D8 light regime. The initial population was reproduced in the laboratory for 2–4 generations before the experiments and maintained with a number of individuals over 1000.

Fresh bean pods were introduced in the containers for the females to lay eggs. After 12 h, the bean pods were collected and placed in polypropylene 30 mL cups with *Ephestia* eggs, assessing emergence daily. Newly hatched (<24 h) nymphs (N1) were individually transferred to 5 mL-plastic vials with a section (2 mm diameter, 20 mm length) of cotton petiole (*Gossypium* spp.) inside to provide moisture and covered by a lid. Sixty individuals were used per prey density: 1, 3, 5, 8, and 10 *Ephestia* eggs per day. Every day, the corresponding new eggs were added, and development and mortality were checked under a stereoscopic microscope (Leica Microsistemas, Hospitalet de Llobregat, Spain). Upon adulthood, the individuals were frozen and the width of the pronotum was measured with an optical micrometer (Leica Microsistemas, Hospitalet de Llobregat, Spain at 50×.

To study the effect of the amount of prey on fecundity, freshly emerged adults (<24 h old) were sexed and 30 pairs of both sexes per diet treatment were isolated in polypropylene 30 mL cups with ventilated lids with a piece of green bean pod end-sealed with paraffin wax as an egg-laying substrate. Well-fed adults from the laboratory colony were used to avoid any carry-over effect from immature stages. The male was removed after an hour once mating took place. Prey density treatments were 0, 2, 5, 8, and 12 *Ephestia* eggs per day. Every day, new food was added, and the bean pod section was replaced, counting the number of eggs oviposited. Fecundity was assessed until day 10 since mating as early fecundity has been reported as a good predictor for lifetime fecundity [17,21,23].

Differences among prey densities in developmental times, survival, adult body size, and early fecundity were analysed using one-way ANOVA. Assumptions of normality and homogeneity of variances were checked prior to the analysis. When significant differences between diets were observed, means were separated using Tukey’s HSD test.

## 3. Results

**Figure 1:**
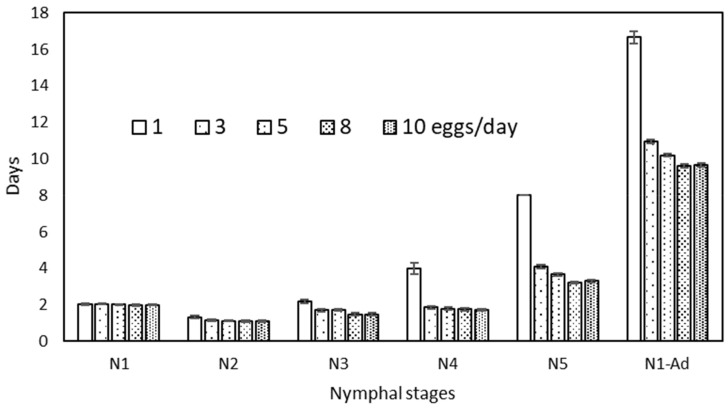
Developmental times (Mean ± SE) of each nymphal stage (N1–5) and nymph to adulthood (N1-Ad) of *Orius laevigatus* when fed different amounts of *Ephestia kuehniella* eggs per day.

The duration of nymphal development of *O. laevigatus* was significantly affected by the amount of *Ephestia* eggs provided per day (Figure 1, Table 1). Individuals supplied with only 1 egg showed the longest developmental time (16.7 ± 0.3 days). In contrast, those supplied with 8 and 10 eggs per day exhibited the shortest duration from N1 to adults (9.6 ± 0.1 and 9.7 ± 0.1 days, respectively), with the treatments of 3 and 5 eggs in an intermediate position (11.0 ± 0.1 and 10.2 ± 0.1 days, respectively). The duration of the youngest nymphal stage was no different among treatments, but the difference among diets increased with age. From second nymphal instar (N2), the treatment with the lowest amount (1 egg/day) showed significantly longer developmental times. The differences were more marked for the last nymphal stage (N5), with developmental times for 1 egg/day more than twice as those observed in the other treatments. The diets with 3 and 5 eggs/day resulted in longer developmental times, especially in the oldest nymphal stage (N5).

**Figure 2:**
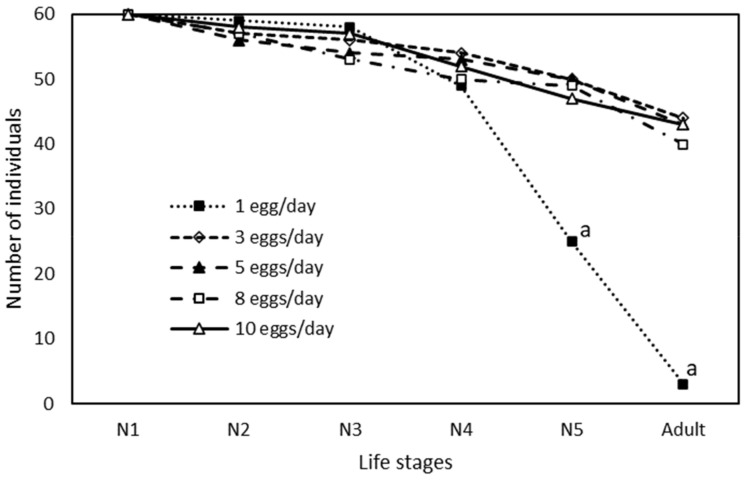
Number of survivors (initial number = 60) along development of each nymphal stage of *Orius laevigatus* when fed different amounts of *Ephestia kuehniella* eggs per day. ^a^ Value is significantly different from the others within the same nymphal stage.

The survival rate was only significantly lower for the diet with the lowest amount of *Ephestia* eggs (1 egg/day) (Figure 2), with a very poor survival from N1 to adult. However, this difference was only significant from N4.

The body size of adults from the different diets was also affected by the amount of prey supplied throughout the immature stages (Table 2). Male body size was significantly higher for the diets consisting of 8 and 10 eggs per day, and the lowest for 1 egg. Female body size was significantly different among all diet treatments, with the largest body size for the diet with the maximum amount of *Ephestia* eggs (10 eggs/day).

**Figure 3:**
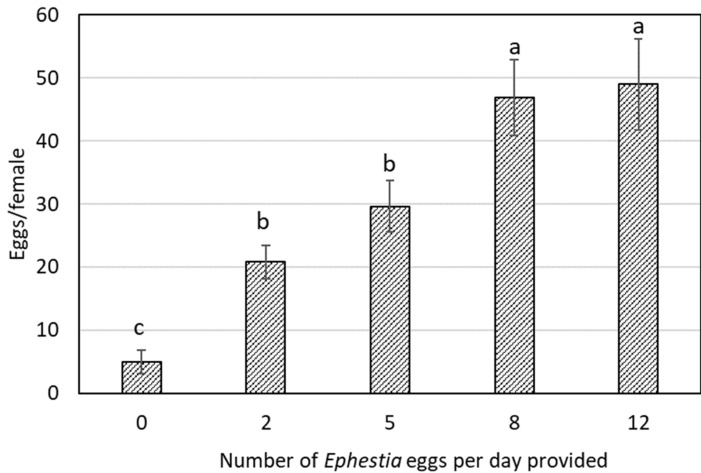
Fecundity of *Orius laevigatus* females in 10 days after emergence (Mean ± SE) when provided different amounts of *Ephestia kuehniella* eggs per day. Bars with the same letter are not significantly different (F_4,57_ = 8.31, *p* < 0.001).

The amount of *Ephestia* eggs per day provided to the adult female impacted fecundity (Figure 3). When provided with no prey, the females showed an average fecundity of only 5.0 ± 1.8 eggs per female. Conversely, the females supplied with 8 and 12 eggs per day exhibited the highest fecundity, laying 46.8 ± 6.0 and 49.0 ± 7.2 eggs per female, respectively.

Regarding the percentage of females laying eggs, only 30% of the females with no prey available were capable to oviposit, a percentage significantly lower than that for the females provided with 5, 8, and 12 eggs (70–80%), with those supplied with 2 eggs in an intermediate position (55%).

## 4. Discussion

Economical mass rearing of natural enemies is pivotal for their use as augmentative biological control agents. To this end, the conditions for mass production have been widely investigated for different natural enemies, including *O. laevigatus*. The key component for cost optimization of production systems is the type and amount of diet supplied. In the case of the *Orius* species, *Ephestia* eggs have proved to be nutritionally superior to other cheaper natural and artificial foods [8,9,10,11,12,13,14,15,16,17,18,19,20]. Consequently, the current practice in industrial production is the use of these eggs as factitious prey. However, as far as we know, there is no published work on the minimal daily food supply for the different life instars of *O. laevigatus*, which should provide essential information for cost-effective mass-rearing.

In our study, at least a daily supply of 1 and 3 eggs of flour moth was needed for optimal development of the first two nymphal instars (N1 and N2), respectively, although for maximum survival, 1 egg per day was enough for both instars. For subsequent development until adulthood, a minimum of 8 eggs per day were needed to fully support growth, but only 3 eggs per day for optimal survival.

A previous work studied the minimum amount of *Ephestia* eggs for development and survival of *O. sauteri* (Poppius) from egg to adult [21]. They recorded a minimum of 7.5 eggs per day (30 *Ephestia* eggs per four days) for optimal developmental rate. Similarly, another study reported a daily supply of 8 *Ephestia* eggs for maximum development and survival of *O. insidiosus* (Say) [22]. Both data are fully consistent with our findings regarding an optimum of 8 *Ephestia* eggs per day for maximum developmental rate of *O. laevigatus* from egg to adult. However, we went further—minimizing costs by lowering the minimum amount for the youngest nymphs with 1 egg/day for N1, 3 eggs/day for N2 and then 8 eggs/day for N3 and ensuing instars.

Considering the duration of the development and the optimal amount of *Ephestia* eggs, 76.8 eggs (8 eggs/day × 9.6 days) will be needed to obtain an adult of *O. laevigatus*. This amount is much lower than that considered standard for *Orius* rearing (216 *Ephestia* eggs) [24], or that obtained as the pre-imaginal predation capacity (174.6 eggs) [25]. However, the minimal amount of prey for maximum growth is expected to be lower than the number of prey an individual is capable to eat. Extracting the data from the respective works, 92 eggs (8 eggs/day × 11.5 days) and 102.7 eggs (7.5 × 13.7) will be required for a female of *O. insidiosus* [22] and *O. sauteri* [21]. This suggests that *O. laevigatus* is expected to be cheaper to produce. For a market price of *Ephestia* eggs (600–800 €/kg) [7], and an average number of 36000 eggs per gram, the cost of the diet for producing *O. laevigatus* will range 1.28–1.71 Euros per 1000 individuals.

However, taking into account the specific needs and duration for each nymphal stage obtained in the present study for *O. laevigatus*, the cost of the food can be optimized: 2.05 eggs for N1, 3.54 for N2, 11.84 for N3, 13.60 for N4, and 25.84 for N5, totalling 56.87 eggs. Therefore, the diet costs would be reduced to 0.95–1.26 € per 1000 females, saving 0.33–0.44 € in every bottle of 1000 *Orius* produced.

*O. laevigatus* nymphs were well adapted to survive with a very limited amount of food. They were able to complete development with a very low prey density. With a food supply of only 1 eggs of flour moth per day, 5% of the nymphs reached adulthood, but with 3 eggs/day 73% of them completed development, as much as with higher availability of *Ephestia* eggs. For *O. insidiosus*, almost 60% of individuals were able to develop from egg to adult with 1 *Ephestia* egg per day [22]. Similarly, in *O. sauteri* with a daily supply of 1.25 eggs, 42% of nymphs reached adulthood [21]. Other omnivorous predator, the mirid *Macrolophus pygmaeus* (Rambur) (Hemiptera: Miridae), a species with larger body size than *Orius*, had a 64% survival in the juvenile development feeding only 1.7 *Ephestia* eggs per day [7].

Moreover, with the lowest food supply (1 egg/day), the mortality from N1 to N3 was not different to superior diets in our study. For *O. insidiosus* it was reported that survival was not reduced in the 3 first days after hatching with a daily diet supply of 1 egg per day [22]. Three days is the duration of N1 and part of N2. Indeed, in our study, survival was maintained until N4, which accounts for around five days after hatching, suggesting a high resilience of young instars facing food shortage and improving the likelihood of finding prey. This ability to survive and complete development under prey scarcity is one of the keys for the successful establishment of *O. laevigatus* and other omnivorous predators when early released on the crop prior to pest outbreak, as well as for continuous presence along the crop cycle despite pest density fluctuations [26].

On the other hand, body size is an important indicator of the quality of a predator as biological control agent, as it has a profound effect on most biological and ecological traits such as fecundity, longevity, predation, resistance to starvation, etc., [27,28,29,30,31]. In addition, adult body size is determined by the nutritional characteristics (quantity and quality) of the diet ingested in immature development [8,9,10,11,12,13,14,15,16,17,18,19,20,31]. As stated above, the minimum amount of *Ephestia* eggs for optimal survival and developmental rate, and for cost-effective rearing, was assessed in 8 *Ephestia* eggs per day. Similarly, male body size was also maximized feeding 8 eggs daily, but for maximum female body size, 10 eggs per day were required. Therefore, given a balanced sex ratio typical of *Orius* species, a daily supply of 9 *Ephestia* eggs will be required for maximizing body size and hence biocontrol efficiency of the adults once released on the crop. Similarly, the amount of *Ephestia* eggs needed to maximize adult body size was higher for females (7.5 eggs/day) than for males (2.5 eggs/day) in *O. sauteri* [21], but equal (8 eggs/day) for both sexes in *O. insidiosus* [22].

Oviposition rate of *O. laevigatus* females increased with the daily number of *Ephestia* eggs provided, until a plateau was reached at 8 eggs/day. This amount is the same as that obtained for *O. insidiosus* [22], a very close to 10 eggs/day for *O. sauteri* (8 eggs/day was not tested, just 5 eggs/day, which also yielded an inferior oviposition in our study) [21]. *Orius* females are able to lay eggs with a very limited food intake, but unable in the absence of nutrition. Although in our study, *O. laevigatus* females laid some eggs when no *Ephestia* eggs were provided, this was rather a residual effect on early reproduction from nutrition during juvenile stages [22,23,31]. *Orius* species are income breeders, which exploit nutritional intake as adults for reproduction, instead of capital breeders, which use resources stored during immature development [23,31,32].

## 5. Conclusions

This study indicates a minimum of 1 and 3 *Ephestia* eggs per day for the first two nymphal instars of *O. laevigatus*, respectively, and 8 eggs per day for subsequent development until adulthood. However, a daily supply of 9 *Ephestia* eggs will be required for maximizing body size and hence biocontrol efficiency of the adults once released on the crop. This information may contribute to the reduction in rearing costs of industrial production, which in turn will favor a wider adoption of biological control as main pest control method. However, further research is warranted to validate these results in large scale production systems and over several generations. In addition, this study confirms the ability of *O. laevigatus* to survive and complete development under prey scarcity, which is crucial for their resilience and continuous presence along the crop cycle, despite pest density fluctuations.

## Figures and Tables

**Table 1:** Developmental times in days (mean ± SE) of the nymphal stages of *Orius laevigatus* provided with amounts of *Ephestia kuehniella* eggs.

Eggs/day	N1		N2		N3		N4		N5		N1–N5	
1	2.05 ± 0.04	a	1.33 ± 0.07	b	2.18 ± 0.10	c	4.00 ± 0.31	b	8.00 ± 0.00	d	16.67 ± 0.33	d
3	2.05 ± 0.03	a	1.18 ± 0.05	a	1.72 ± 0.08	b	1.86 ± 0.06	a	4.09 ± 0.08	c	10.95 ± 0.11	c
5	2.02 ± 0.02	a	1.13 ± 0.05	a	1.72 ± 0.07	b	1.80 ± 0.10	a	3.67 ± 0.08	b	10.19 ± 0.08	b
8	1.98 ± 0.03	a	1.11 ± 0.05	a	1.48 ± 0.07	a	1.76 ± 0.07	a	3.23 ± 0.07	a	9.60 ± 0.10	a
10	2.00 ± 0.02	a	1.12 ± 0.04	a	1.48 ± 0.07	a	1.72 ± 0.07	a	3.30 ± 0.08	a	9.67 ± 0.10	a
	F_4,282_ = 0.99 ns		F_4,273_ = 2.79 *		F_4,253_ = 13.56 ***		F_4,216_ = 52.43 ***		F_4,168_ = 75.73 ***		F_4,168_ = 105.8 ***	

Means followed by the same letter in the columns are not significantly different. ns: no significant, * *p* < 0.05, *** *p* < 0.001.

**Table 2:** Adult size (mean ± SE) (number of individuals in brackets) of *Orius laevigatus* after nymphal development feeding on different amounts of *Ephestia kuehniella* eggs.

Eggs/day	Female size		Male size	
1	0.585 ± 0.015 (2)	e	0.550 (1)	d
3	0.684 ± 0.005 (21)	d	0.653 ± 0.005 (23)	c
5	0.735 ± 0.005 (21)	c	0.685 ± 0.004 (22)	b
8	0.752 ± 0.006 (20)	b	0.736 ± 0.005 (20)	a
10	0.768 ± 0.005 (26)	a	0.735 ± 0.004 (17)	a
	F_4,85_ = 57.2 ***		F_4,78_ = 70.8 ***	

Means followed by the same letter in the columns are not significantly different. *** *p* < 0.001.

## Data Availability

The datasets generated and/or analysed during the current study are available from the corresponding author on reasonable request.
